# Membrane Translocation of IL-33 Receptor in Ventilator Induced Lung Injury

**DOI:** 10.1371/journal.pone.0121391

**Published:** 2015-03-27

**Authors:** Shih-Hsing Yang, Jau-Chen Lin, Shu-Yu Wu, Kun-Lun Huang, Fang Jung, Ming-Chieh Ma, Guoo-Shyng Wang Hsu, Guey-Mei Jow

**Affiliations:** 1 Department of Respiratory Therapy, Fu Jen Catholic University, New Taipei City, Taiwan; 2 P.h.D Program in Nutrition and Food Science, Fu Jen Catholic University, New Taipei City, Taiwan; 3 Institute of Aerospace and Undersea Medicine, National Defense Medical Center, Taipei, Taiwan; 4 School of Medicine, Fu Jen Catholic University, New Taipei City, Taiwan; 5 Department of Nutritional Science, Fu Jen Catholic University, New Taipei City, Taiwan; French National Centre for Scientific Research, FRANCE

## Abstract

Ventilator-induced lung injury is associated with inflammatory mechanism and causes high mortality. The objective of this study was to discover the role of IL-33 and its ST2 receptor in acute lung injury induced by mechanical ventilator (ventilator-induced lung injury; VILI). Male Wistar rats were intubated after tracheostomy and received ventilation at 10 cm H2O of inspiratory pressure (PC10) by a G5 ventilator for 4 hours. The hemodynamic and respiratory parameters were collected and analyzed. The morphological changes of lung injury were also assessed by histological H&E stain. The dynamic changes of lung injury markers such as TNF-α and IL-1β were measured in serum, bronchoalveolar lavage fluid (BALF), and lung tissue homogenization by ELISA assay. During VILI, the IL-33 profile change was detected in BALF, peripheral serum, and lung tissue by ELISA analysis. The Il-33 and ST2 expression were analyzed by immunohistochemistry staining and western blot analysis. The consequence of VILI by H&E stain showed inducing lung congestion and increasing the expression of pro-inflammatory cytokines such as TNF-α and IL-1β in the lung tissue homogenization, serum, and BALF, respectively. In addition, rats with VILI also exhibited high expression of IL-33 in lung tissues. Interestingly, the data showed that ST2L (membrane form) was highly accumulated in the membrane fraction of lung tissue in the PC10 group, but the ST2L in cytosol was dramatically decreased in the PC10 group. Conversely, the sST2 (soluble form) was slightly decreased both in the membrane and cytosol fractions in the PC10 group compared to the control group. In conclusion, these results demonstrated that ST2L translocation from the cytosol to the cell membranes of lung tissue and the down-expression of sST2 in both fractions can function as new biomarkers of VILI. Moreover, IL-33/ST2 signaling activated by mechanically responsive lung injury may potentially serve as a new therapy target.

## Introduction

Ventilator-induced lung injury (VILI) has been recognized as a form of acute lung injury directly induced by mechanical ventilation [1–3]. The manifestations of VILI as related to alveolar pathophysiology consist of air leaks, increased endothelial and epithelial permeability, and the release of inflammatory mediators [4,5]. Despite the suggestion of previous studies that a lung protective ventilator strategy should be used to prevent the problems associated with VILI, the mortality rate due to VILI has remained high at between 30% and 63% [6,7]. Therefore, further understanding of the inflammatory mechanisms of VILI is of critical importance.

A previous study found VILI to be associated with significantly increased levels of IL-1α, IL-1β, TNF-α, IL-6, and IL-10 in the lung tissue homogenate [8]. Inflammatory cytokines enter the bloodstream and bronchoalveolar lavage fluid (BALF) and may cause adverse inflammatory reactions in systemic tissues, thereby increasing mortality [1,9,10]. However, the expression of these inflammatory cytokines can be modified by ventilator management [10]. It has previously been demonstrated that a lung-protective strategy could reduce concentrations of these inflammatory cytokines in BALF as well as in plasma [10].

IL-33 is a newly identified pro-inflammatory cytokine of the IL-1 family that is a ligand for the orphan IL-1 family receptor ST2 [11]. IL-33 is reported to be involved in pulmonary diseases such as severe asthma and immunopathological diseases such as arthritis [12]. The transmembrane ST2 (ST2L) and soluble ST2 (sST2) isoforms act as the receptors for IL-33. The ST2L form is necessary for the extracellular effects of IL-33 as IL-33 binds to receptor complexes composed of ST2L to induce a pro-inflammatory Th2-associated cytokine response [13,14]. The circulating sST2 form has been reported as an important predictor in patients with heart failure[15,16], acute dyspnea [17] and myocardial infarction [18].

IL-33 is broadly expressed in various tissues, including epithelial cells lining bronchial and lung tissues [14,19]. IL-33 is also expressed as a mechanically responsive cytokine secreted by living cells. In a previous study, fibroblasts received mechanical strain for 8 hours; the concentration of extracellular IL-33 was significantly increased during the first 4 hours and then subsequently declined [19]. In another previous study showed both ST2L and sST2 were also induced by biomechanical properties in cardiomyocytes [20]. Despite IL-33 being secreted as a response to mechanical stimulus, the profile of IL-33 expression during mechanical ventilation is not clearly understood. Therefore, whether IL-33/ST2 signaling may be used as a biomarker of VILI in animal models remains unclear. The purpose of this study was to demonstrate that IL-33 is a novel biomarker associated with VILI. We used a mechanical ventilator to establish high-pressure ventilation in a rat model to examine the mechanisms of VILI and thereby identify potential clinical strategies.

## Materials and Methods

### Animals and surgical preparation

Male Wistar rats were obtained from a provider of rats for animal experiments (BioLASCO CO., LTD, Taipei, Taiwan); rats weighing between 220 and 300 g were used. All the protocols were approved by the Laboratory Animal Care Committee of Fu-Jen Catholic University. All animals were handled in accordance with the Guide for the Care and Use of Laboratory Animals [21]. Each rat was anesthetized with a mixture of 20–40 mg/kg of Zoletil 50 (Vibac Laboratories, Carros, France) and 5–10 mg/kg of Rompun (Bayer, Leverkusen, Germany) injected into the intraperitoneal space [22]. Systemic mean arterial blood pressure (MABP) and heart rate (HR) were continuously monitored through acquisition system instruments (MP 100, BIOPAC system Inc.) and recorded via computer (Chart 4 record system; Biopack Systems Inc., Santa Barbara, CA). In addition, a cannula was inserted into the trachea [5,23] and a G5 ventilator (Hamilton Medical AG, Switzerland) was used and connected to an end tidal expiratory CO_2_ monitor (PETCO_2_; Hamilton Medical AG, Switzerland).

### Experiment protocol

Animals were divided into two groups, a control group (CTL; meaning no mechanical ventilator was used) and an inspiratory pressure at 10 cmH_2_O group (PC10). The animals in the latter group were ventilated at the pressure control mode setting in a G5 ventilator, including inspiratory pressure at 10 cmH_2_O, respiratory rate of 50 beat/min, an inspiratory time of 0.3 seconds, positive end-expiratory pressure (PEEP) at 0 cm H_2_O, and a inspiratory oxygen fraction (FiO_2_) of 21%. Ventilator intervention was then performed on each animal for 4 hours, during which data was collected.

### Measurement of arterial blood gas

Arterial blood was collected from the left femoral artery at each hour. Using a portable blood gas analyzer (i-STAT, Princeton, NJ), we performed measurements of the blood samples, including measurements of arterial oxygen tension (PaO_2_), carbon dioxide tension (PaCO_2_), acidity (pH) and O2 saturation (SaO_2_).

### Histopathological analysis of lung tissues

The lung lobe was stained with hematoxylin and eosin to analyze the histopathological changes in the lung. The lung injury score was examined within each field by microscopy, and lung injury was scored according to (A) the infiltration or aggregation of neutrophils in the airspace or vessel wall, and (B) the thickness of the alveolar wall. Each assessment yielded a grade of 0, 1, 2, or 3, for no, mild, moderate, or severe injury, respectively [24]. The resulting two scores were added and presented as the lung injury score. In BALF analysis, bronchoalveolar lavage fluid (BALF) samples were obtained after each rat’s lung was removed and tied on the right side, as in a previous study [25]. The cell pellets and supernatants of BALF were separated by centrifugation (1000 rcf). The supernatants of BALF were stored in a refrigerator for ELISA assay. The protein concentration in BALF was determined using the bicinchoninic acid (BCA) protein assay kit (Thermo scientific, Rockford, IL, USA). The cell pellets were resuspended in the PBS buffer (100μL), and then were stained with Türk solution (Wako Pure Chemical Industries Ltd.). The number of nucleated cells that stained white cells were counted using a light microscope (Olympus Optical, Tokyo, Japan).

### Measurement of cytokine levels

TNF-α, IL-1β, and IL-33 were obtained for measurement using a standardized sandwich enzyme-linked immunosorbent assay (ELISA) kit according to the manufacturer’s instructions (R&D system) [26]. The absorbance was read at 450 nm using an Epoch ELISA reader (Epoch, BioTek, USA).

### Western blotting analysis

Immunoblotting was performed as described previously [27]. The cytoplasmic, nuclear, and mitochondrial proteins of lung tissues were extracted by using a Nuclear/Cytosol Extraction Kit and a Mitochondria/Cytosol Fractionation Kit (BioVision, Inc., Mountain View, CA, USA) according to the manufacturer’s instructions. The 30 μg/lane equal amounts of lung homogenates were fractionated and subjected to 10–12% sodium dodecyl sulfate-polyacrylamide electrophoresis (SDS-PAGE), transferred to polyvinylidene fluoride membranes, and immunoblotted with polyclonal antibodies against IκB-α, P-NF-κB p65, NF-κB p65 (1:1000 dilution; Cell Signaling Technology, Danvers, MA, USA), IL-33 (1:100 dilution; Santa Cruz Biotechnology, Santa Cruz, CA, USA), ST2 (1:200 dilution; Bioss, Woburn, MA, USA), and β-actin (for cytoplasmic proteins, diluted 1:10,000; Sigma).

### Immunohistochemistry stain

Lung tissues in formalin-fixed paraffin sections (4-μm) were stained with hematoxylin and eosin (H&E, X 400) [5,23]. In addition, unstained sections were deparaffinized before antigen retrieval and endogenous peroxidase was blocked using 3% H_2_O_2_ in methanol for 15 min. The slides were then incubated for 60 min with a polyclonal antibody against ST2 (1:100 dilution; Bioss, Woburn, MA, USA). After washing, slides were sequentially incubated with rat tissue specific horseradish peroxidase-polymer anti-rabbit antibody (Nichirei Corporation, Tokyo, Japan) for 30 min. The horseradish peroxidase was then reacted with diaminobenzidine substrate for 3 min, and the sections were then counterstained with hematoxylin.

### Statistical Analysis

Numeric data are presented as the mean±SEM. Statistical comparisons between groups were performed using ANOVA and Bonferroni post hoc test. Differences were regarded as significant at values of P < 0.05.

## Results

### Induced lung injury in high pressure ventilation

The VILI rat model was established by using a clinical ventilator. The characteristics of the physiology were recorded (Table 1). The tidal volume (V_T_) of the PC10 group was around 16–18 mL/kg. The end-tidal CO_2_ (PETCO_2_) was mildly reduced, but there was no significant difference. In addition, the hemodynamic system in terms of mean arterial blood pressure and heart rate also exhibited no significant differences during the 4 hours of the experiment.

**Table 1:** The observation change of hemodynamic and respiratory parameters in VILI.

		CTL		PC10				
Weight _(g)_		252.7±13.7		275.8±11.5				
		Baseline	Baseline	0h	1h	2h	3h	4h
meanABP _(mmHg)_		89.2±17	89.4±2.7	75.7±4.6	75.5±4.1	75±3.9	88.2±5.2	84.9±4.1
Heart rate _(/min)_		241.1±30	261.2±22	237.3±32.4	250.6±38.3	231.5±31.4	282.5±25	277.4±21
ABG								
	pH	7.38±0.0	7.37±0.02		7.32±0.02	7.25±0.04	7.28±0.04	7.26±0.06
	PaCO_2 (mmHg)_	49.4±2.1	53.3±1.6		59.7±3.5	61.3±6.3	65.0±6.3	60.4±11.4
	PaO_2 (mmHg)_	75.7±9.1	76.3±5.0		94.9±11.3	98.2±15.1	95.1±6.6	95.1±6.6
	HCO3^-(mmol/L)^	29.3±1	30.6±0.4		29.8±1.1	24.7±6.0	30.3±1.3	24.3±4.2
	SaO_2 (%)_	93.3±2.2	93.5±2.5		97.5±0.5	96.0±2.0	96.0±.0.0	96.0±3.0
V_T (mL)_			4.5±0.3		4.9±0.9	4.7±0.9	5.1±1.0	5.0±1.1
PETCO_2 (mmHg)_			31.0±1.7		30.6±2.4	29.3±2.8	29.4±2.4	28.5±1.6

Rats were ventilated with high pressure at 10 cmH_2_O for 4 hours, with n = 8 rats per group. The data are presented by mean±SEM. CTL: control group animals without ventilator administration; PC10: animals ventilated with pressure control mode at 10 cm H_2_O. meanABP: mean arterial blood pressure; ABG: arterial blood gas, V_T_: tidal volume; PETCO_2_: End-tidal CO_2_. The comparison between animals without ventilation and animals receiving ventilation at 10 cmH_2_O showed no significant differences at baseline, and animals in PC10 group also exhibited no significant differences during the 4 hours of the experiment.

**Fig 1:**
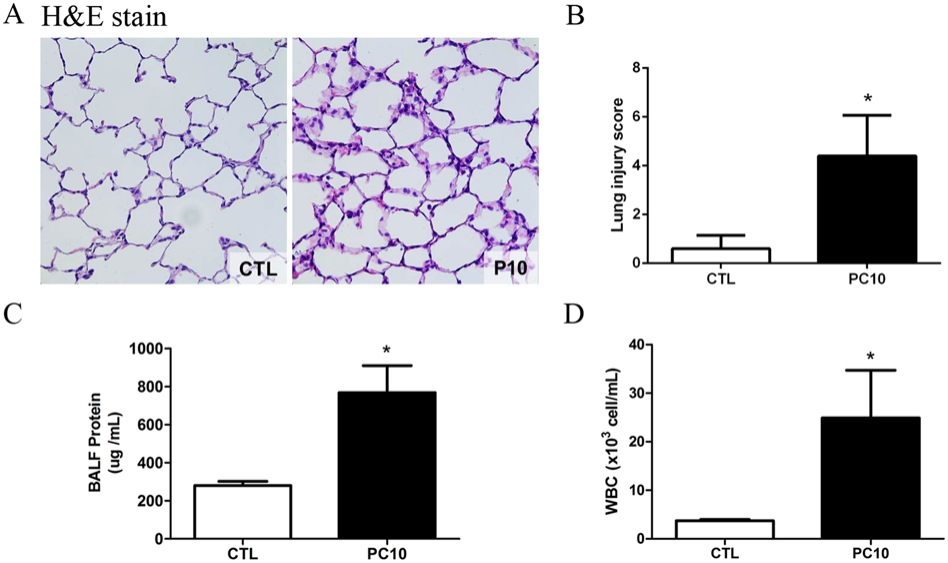
The severity of lung injury was assessed after high-pressure ventilation for 4 hours in each rat. A: Histology of lung tissue was assessed by hematoxylin and eosin staining. Lung tissue sections are shown by a 400× magnification of representative micrograph. Blue staining indicates nuclei counterstain. PC10: animals ventilated with high pressure at 10 cmH_2_O; CTL: control group animals without ventilator administration B: The lung injury score was used to represent the severity of lung injury, and was calculated as the sum of the two scores for alveolar wall thickness and neutrophil infiltration per high power field (400× magnification). C: Protein levels in bronchoalveolar lavage fluid were estimated by BCA kit assay. BALF: bronchial alveolar lavage fluid; D: The numbers of white blood cells in BALF stained with Türk solution were calculated by cell counter under light microscope. *:P <0.001 versus animal without ventilator. The data are presented in terms of mean±SEM for the eight rats in each group.

After 4h of high-pressure ventilation, histological changes including larger alveolar space, thinner alveolar wall, inflammatory cells, lung edema, hyaline membrane and marked alveolar hemorrhaging were evident in the PC10 group (Fig. 1A). However, there were no morphological changes in the control group. The lung injury score was utilized as a measure of lung injury severity, and the average score of the PC10 group was significantly increased compared to that of the control group (Fig. 1B). The BALF protein level in the PC10 group was significantly increased in comparison to the level in the control group (Fig. 1C). Moreover, the numbers of white cells in BALF were also significantly increased in the PC10 group (Fig. 1D).

### Production of inflammatory cytokines

**Fig 2:**
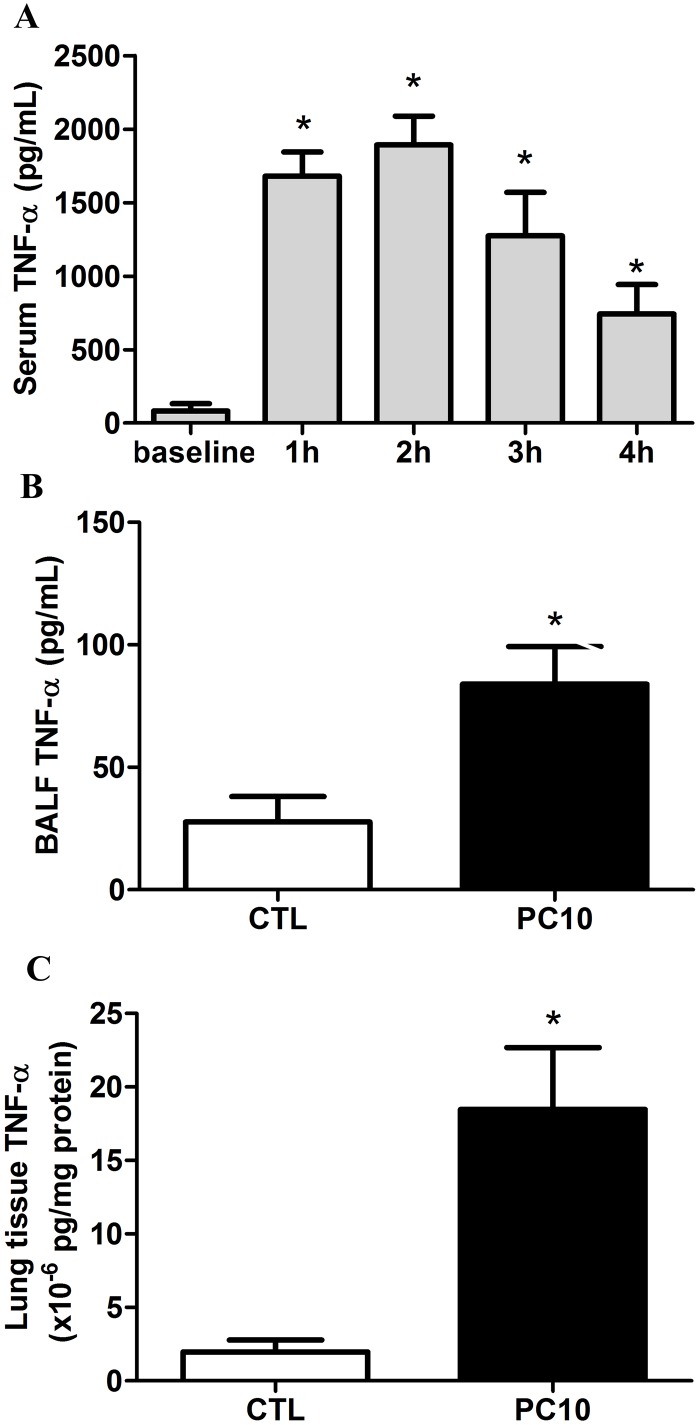
The TNF-α cytokine expression in animals exposed to VILI. A: in serum, B: in BALF, C: in lung tissue homogenization. The cytokine profiles of TNF-α from collected serum, BALF, and lung tissue homogenization samples, respectively, were shown by ELISA kit assay A: Time course of TNF-α in serum during animals exposed to 10 cmH_2_O pressure of ventilation. B and C: The expression of TNF-α in BALF and lung tissue homogenization collected in animals exposing to 10 cmH_2_O of ventilation pressure for 4 hours. PC10: animals exposed to VILI via high pressure ventilation at 10 cmH_2_O for 4 hours, CTL: control group animals without ventilator administration. N = 8 per group.*:P <0.05 compared with baseline point or animal without ventilator. The results are presented in terms of mean±SEM.

Our results showed that the serum TNF-α level was dramatically increased after 1h and 2h of rats receiving high pressure ventilation, and then gradually decreased at 3h and 4h, as compared to the baseline level (Fig. 2A). The TNF-α levels in BALF and lung tissue homogenization were significantly increased in the PC10 group treated with 4h of high-pressure ventilation (Fig. 2B, 2C).

**Fig 3:**
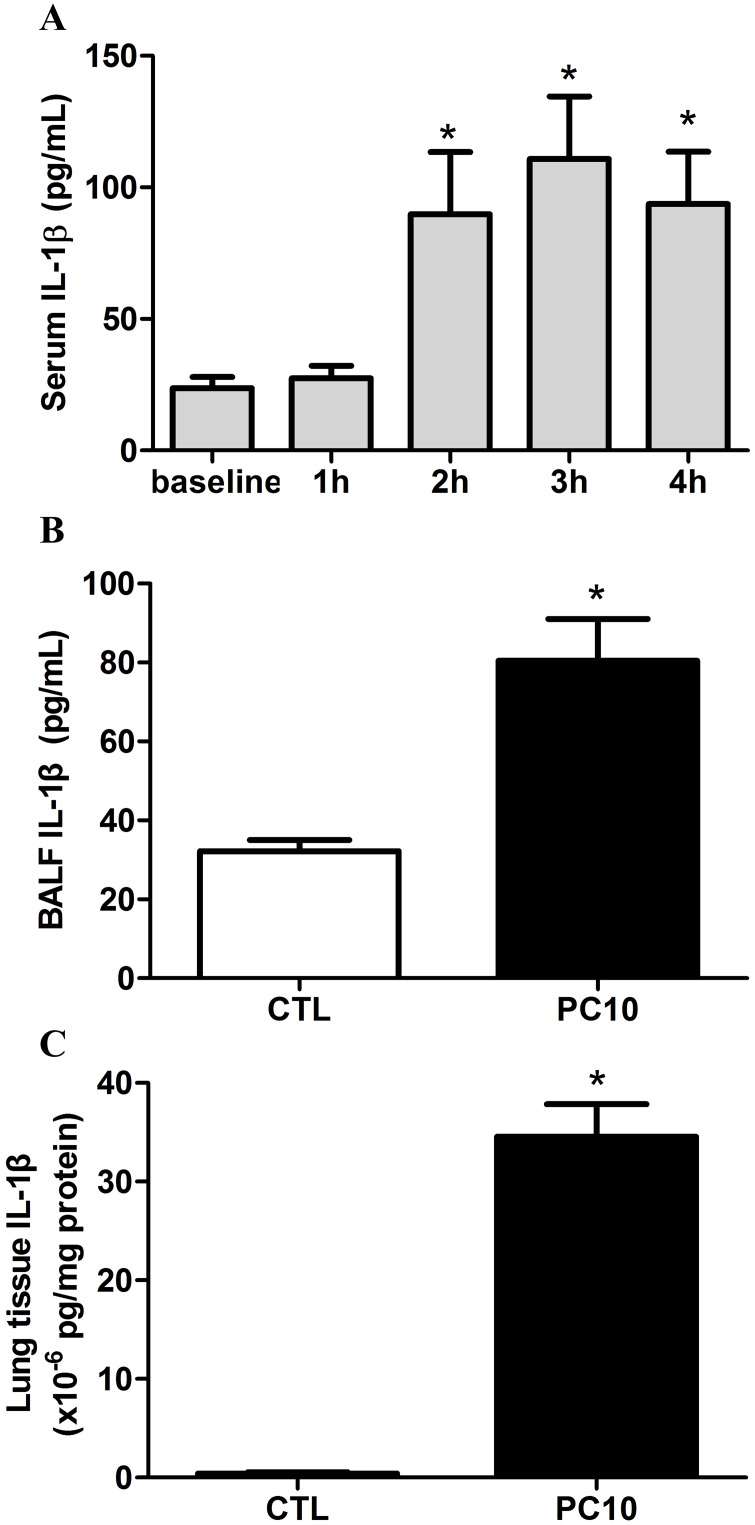
The IL-1β cytokine response in animals exposed to VILI. A: in serum, B: in BALF, C: in lung tissue homogenization. The cytokine profiles of IL-1β from collected serum, BALF, and lung tissue homogenization samples, respectively, were shown by ELISA kit assay. A: Time course of IL-1β in serum, in animals exposed to 10 cmH_2_O pressure of ventilation. B and C: The expressions of IL-1β in BALF and lung tissue homogenization collected in animals exposed to 10 cmH_2_O of ventilation pressure for 4 hours. PC10: animals exposed to VILI via high pressure ventilation at 10 cmH_2_O for 4 hours, CTL: control group animals without ventilator administration. N = 8 per group, *: P <0.05 compared with baseline point or to animals without ventilator. The results are presented in terms of mean±SEM.

The response of IL-1 family cytokines was evaluated in the animal with VILI model. The results indicated that the serum IL-1β was rapidly elevated after 2 h of high-pressure ventilation (Fig. 3A). After 4 h of high-pressure ventilation, the IL-1β levels in BALF and lung tissue homogenization were significantly increased (Fig. 3B-3D).

### Elevated IL-33 response

**Fig 4:**
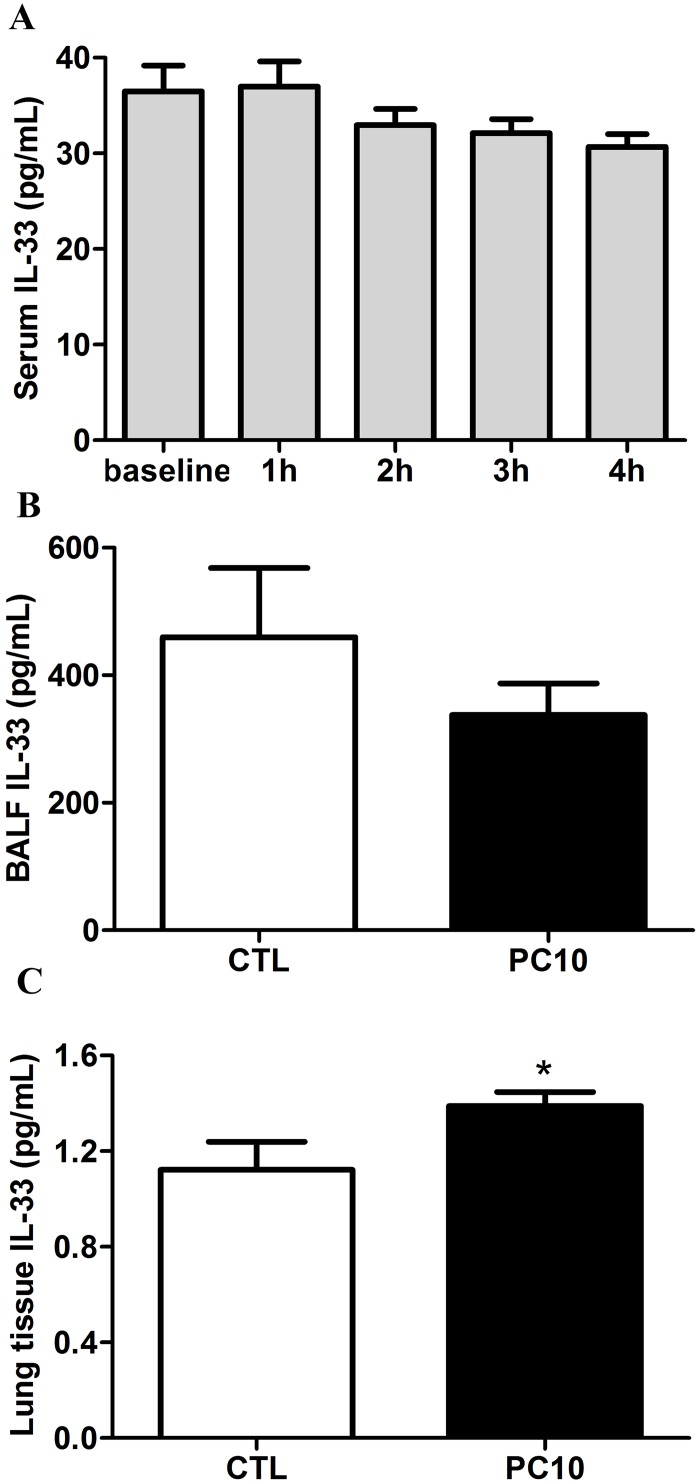
The new IL-33 cytokine level was examined from various sources of rat in VILI. A: in serum, B: in BALF, C: in lung tissue homogenization. The collection samples were determined to IL-33 level by ELISA kit assay. A: Time course of IL-33 in serum in animals exposed to 10 cmH_2_O pressure of ventilation. B and C: The expressions of IL-33 in BALF and lung tissue homogenization collected from animals exposed to 10 cmH_2_O of ventilation pressure for 4 hours. PC10: animals exposed to VILI via high pressure ventilation at 10 cmH_2_O for 4 hours, CTL: control group animals without ventilator administration. *:P <0.05 compared with animals no using ventilator, n = 8. The results are presented in terms of mean±SEM.

**Fig 5:**
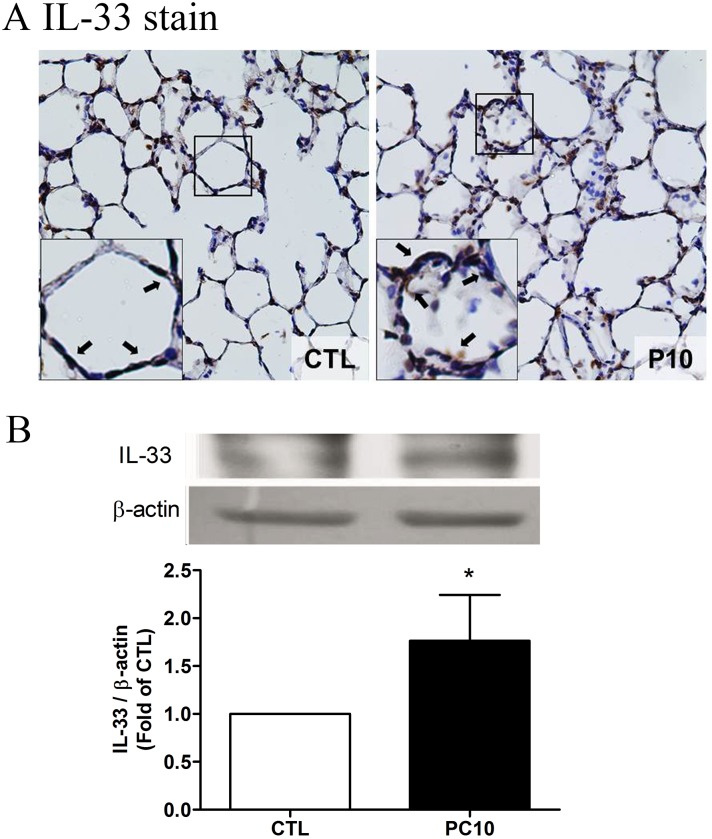
The IL-33 expressed in lung tissue in animals exposed to high pressure ventilation for 4 hours. A: The IL-33 expression of lung tissue sections was determined using immunohistochemistry analysis and colorimetric detection with DAB (brown stain). The figure is presented at a magnification of 400X (insert, 1000X). The arrows reveal that IL-33 staining was remarkably accumulated in the alveolar wall. The slides were incubated using rabbit anti-IL-33 polyclonal antibody. B: IL-33 expression of lung tissue homogenization was determined using western blot assay and densitometry analysis. PC10: animals exposed to ventilation at 10 cmH_2_O for 4 hours, CTL: control group animals without ventilator administration.*:P <0.05 compared with animals without ventilator, n = 8. The results are presented in terms of mean±SEM.

To evaluate whether IL-33 is involved in VILI-induced lung injury, the levels of IL-33 were examined by ELISA assay, immunohistochemistry staining and Western blot analysis. The results showed that the levels of IL-33 were not significantly different in the systemic circulation and BALF (Fig. 4A, 4B). However, the expression of IL-33 in lung tissue homogenization was increased in the PC10 group as compared with the control group (Fig. 4C). Furthermore, the IL-33 protein expression was significantly increased in the PC10 group (Fig. 5). At that magnification, the brown color of IL-33 immunostaining in the alveolar walls of the PC10 group rats indicated higher IL-33 accumulation than in the control group rats (Fig. 5A). And, the IL-33 protein expression was significantly increased in the PC10 group compared to the control group.

### Membrane translocation of ST2 receptor

**Fig 6:**
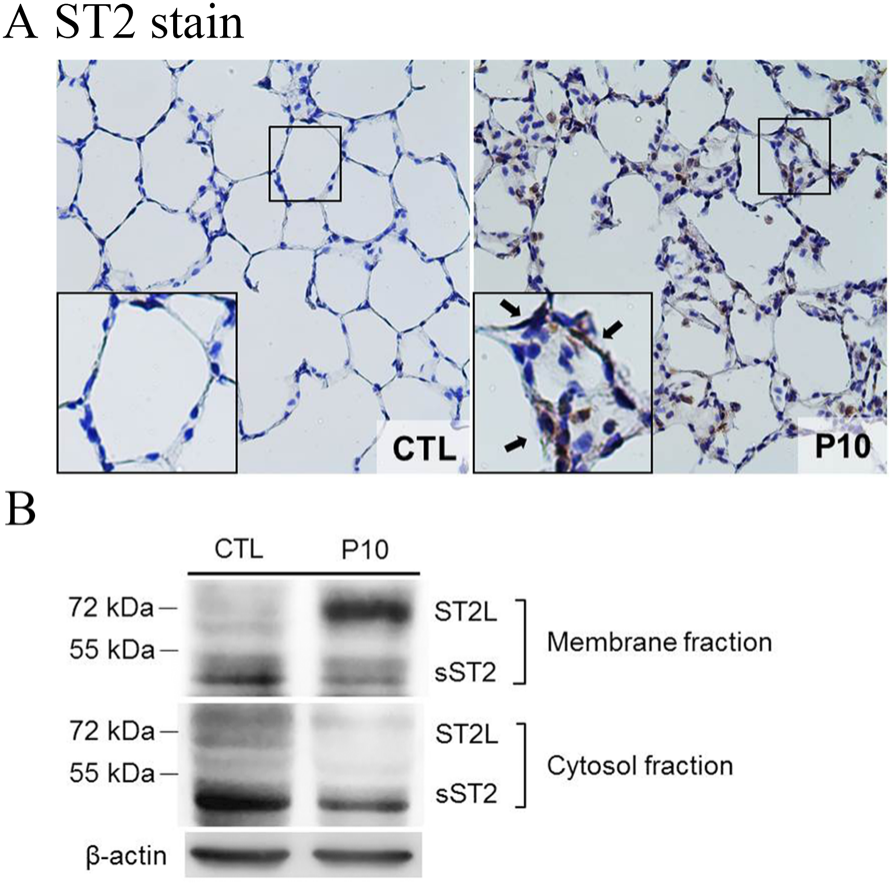
ST2 receptor translocated to cell membranes in lung tissue of rat during VILI. A: The ST2 expression in lung tissue sections was analyzed by using IHC staining with rabbit anti-ST2 polyclonal antibody conjugated DAB (brown stain). The figure is shown at a magnification of 400X (insert, higher magnification of 1000X). The arrows indicate high accumulation of ST2 immunostaining in the alveolar wall. PC10: animals exposed to ventilation at 10 cmH_2_O for 4 hours, CTL: control group animals without ventilator administration. B: Lung tissue homogenization samples were separated into membrane and cytosol fractions and then subjected to western blot assay.

To determine whether the ST-2 protein and IL-33 receptor affected the mechanism of the VILI lung injury pathway, the ST2 expression in lung tissues was examined by immunohistochemistry staining. The results showed that the ST2 expression was dramatically increased in the PC10 group (Fig. 6A). The brown color of ST2 revealed by the immunostaining of lung tissues indicated that ST2 accumulation in the alveolar walls of the PC10 group animals was much higher than in the control group animals (Fig. 6A).

To identify the feature of ST2L and sST2 expression, the lung tissue homogenization was then analyzed by Western blot analysis. The results showed that ST2L was highly accumulated in the membrane fraction of lung tissue in the PC10 group, but that the ST2L in cytosol was dramatically decreased in the PC10 group compared to the control group (Fig. 6B). In addition, sST2 was slightly decreased in the membrane and cytosol fractions of the PC10 group when compared to the control group.

### Increased NF-κB expression

**Fig 7:**
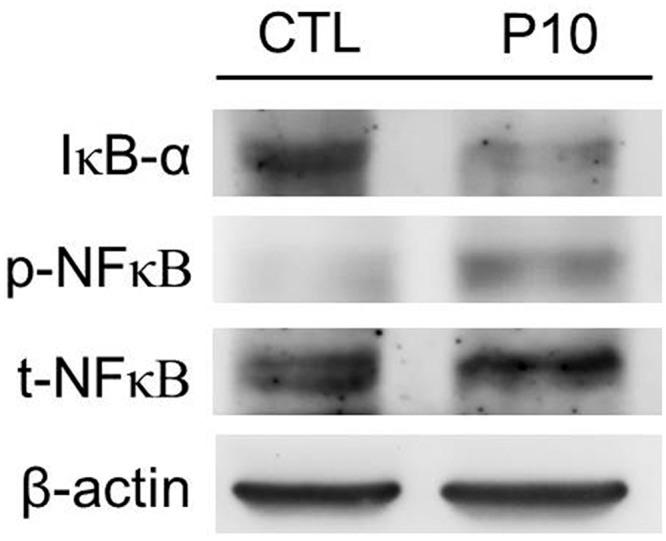
The NF-κB pathway of lung tissue in animals subjected to VILI. Densitometry analysis of the IκB-α, p-NF-κB, and t-NF-κB levels expressed in western blot assay performed using rabbit anti-IκB-α, p-NF-κB, and t-NF-κB polyclonal antibodies, respectively, in lung tissue homogenization samples. PC10: animals exposed to ventilation at 10 cmH_2_O for 4 hours, CTL: control group animals without ventilator administration.

We further investigated whether NF-κB activation is involved in IL-33-mediated VILI. As shown in Fig. 7, the phosphorylation of NF-κB was significantly increased in PC10 group. But the IκB-α protein, as a bound protein of NF-κB, was decreased in the PC10 group after 4 h of high-pressure ventilation.

## Discussion

In this study, we demonstrated that IL-33 receptors in the alveoli could function as a new biomarker of biomechanical response to ventilator stimulation. High-pressure ventilation increased the inflammatory response of IL-33, and its receptor ST2 in lung was excessively accumulated. Moreover, ST2L-induced membrane translocation resulted in VILI in rats. These findings suggest that an IL-33/ST2 pathway can contribute to VILI and, furthermore, may have a protective effect on lungs.

Mechanical ventilation may lead to serious lung damage. Numerous studies of animals and human models of VILI have demonstrated that overdistension/stretching or cyclical recruitment/derecruitment of alveoli/small airways leads to significant pulmonary pathophysiology consequences including: leukocyte sequestration, air leaks, increased endothelial and epithelial permeability, infiltration or aggregation of neutrophils in the airspace or vessel wall, thickness of the alveolar wall, and the release of inflammatory mediators [4,5]. VILI intensity has previously been shown to be determined by the duration and strain/stress of mechanical ventilation [1,28]. However, the advantage in this study’s design was that it used a rat model subjected to a medical G5 ventilator with a pressure control mode that mimicked intensive critical care. Rats were ventilated for 4 h in a pressure-controlled mode at 10 cmH_2_O of inspiratory pressure, resulting in a ventilation tidal volume of around 16–17 mL/kg. This tidal volume (V_T_) ventilation of was commonly achieved the in VILI animal model. The determining hemodynamic and gas exchange was also important due to may modulate VILI in the initial experiment design. Our results showed that the mean arterial blood pressure trend during VILI was unchanged. This blood pressure result was consistent with that of a previous study which observed a VILI animal model with a VT~ 22 mL/Kg and respiratory rate of 90 breathes/minute for 4 hours [28]. In this study, gas exchange measurements indicated normal oxygenation and stable CO_2_ elimination. This is consistent with previous animal studies regarding gas exchange in the context of VILI [5,28]. However, pathophysiological imaging of the present study showed alveolar permeability changes, increased lung injury scores, increased total protein concentrations and PMN counts in BALF in animals with VILI, findings which are all consistent with the pathophysiology of VILI found in previous studies [5,29,30].

In addition, two categories of cytokine levels were demonstrated to contribute to the inflammatory responses of VILI [31]. Type I cytokines such as TNF-α and IL-1β are considered the predominate regulators of innate immunity and early inflammation by promoting cytotoxic T cell responses and delayed type hypersensitivity. Type II cytokines such as IL-6 and IL-10 are considered the predominate regulators of the humoral and fibroproliferative responses. The injurious strategies of mechanical ventilation lead to the release of TNF-α and IL-1β, causing increased VILI. A previous study by Nin et al. that subjected Sprague-Dawley rats to 75 minutes of high V_T_ at 35 mL/kg, 70 breaths/min, and FiO_2_ 0.35 found that the release of systemic cytokines, including TNF-α, IL-6, lactic acid, and AST, from the lungs was significantly increased [5]. Dolinay et al. recently investigated VILI under the condition of 8 hours with a V_T_ of 12 mL/kg and observed increases IL-1β levels in lung tissue [26]. In the present study, the consequences of VILI also included significantly increased TNF-α and IL-1β in lung tissue. Moreover, the BALF and serum levels of TNF-α and IL-1β in VILI were significantly higher compared to the CTL group. The consequences of the present study were also similar to those of a recent animal VILI study using a clinical ventilator conducted by Xia et al to examine the profile of cytokines in an animal VILI model. They used the Drager Evita 4 clinical ventilator and the VILI of high V_T_ was 10–15 mL/kg. The BALF and serum levels of rabbits with VILI were analyzed and showed increased levels of TNF-α, IL-1β, IL-6 and IL-10, respectively [32]. Therefore, the expression of TNF-α and IL-1β is generally indicated as being germane to the pathogenesis of VILI.

Interleukin-33 was originally identified in 2003 by Girard and colleagues as a member of the IL-1 family of cytokines [33]. Numerous of studies subsequently demonstrated that IL-33 took the role of a pro-inflammatory cytokine. Schmitz et al. found that IL-33 potently drives the production of pro-inflammatory Th2-associated cytokines, including IL-4, IL-5, and IL-13, by in vitro polarized Th2 cells, the IL-33 production of pro-inflammatory Th2-associated cytokines was via the ST2 receptor [11]. ST2 is a receptor for IL-33, an orphan receptor. ST2 exists in two forms, soluble (sST2) and membrane bound (ST2 ligand: ST2L), both of which bind IL-33 [11,34,35]. IL-33 is known to signal via an ST2L and interelukin-1 receptor accessory protein, activating downstream tyrosine kinases and modulating NF-κB activity [11,36]. A previous study revealed that soluble ST2 directly binds IL-33 and further suppresses the activation of NF-κB in EL-4 cells, which suggests that soluble ST2 may sometimes act as a decoy receptor [37]. Despite the fact that IL-1β and TNF-α are elevated in most VILI but not all [8], the involvement of other potent pro-inflammatory cytokines in the VILI mechanism is still being debated.

This study mainly investigated whether the newly identified IL-33/ST2 pathway is involved in VILI. Previously, Kakkar et al. reported pro-inflammatory IL-33 as a mechanically responsive cytokine [19]. They found that over the first 4 hours of a cyclic strain at 1Hertz, with an 8% biaxial stretch in a fibroblast cell line, the extracellular concentration of IL-33 was gradually and significantly increased, whereas at the 8^th^ hour of strain rapid degradation occurred. Sanada et al. also found that, when placed under cyclic biomechanical strain (8%, 1 Hz), the gene expressions of IL-33 and sST2 were more than 5-fold greater in cardiac fibroblasts than in rat neonatal cardiomyocytes [20]. Therefore, the findings above indicate that IL-33/ST2 signaling plays a role in inflammation mediation, in addition to being a mechanism known to be involved in biochemical stress.

However, the IL-33/ST2 activation can induce lung injury. The findings of Le Goffic et al. evidenced a profound expression of IL-33 in lungs during both in vivo and in vitro influenza A virus infections; a significant increase in the mRNA expression of IL-33 in infected mice compared with noninfected mice was observed [12]. This finding suggests a role for IL-33 involving the virus-induced lung infections. Akiko et al also found that IL-33 mediates inflammatory responses via the ST2 receptor in human lung tissue cells [38]. The IL-33/ST2 pathway causes production of IL-8, IL-6 and ERK, p38 phosphorylation. With regard to mechanical stress, a study by Dolinay et al. indicated that inflammasome-regulated cytokines are critical mediators of acute lung injury [26]. The cytokines measured in that study included IL-18, IL-1β, and IL-33. Part of their results IL-33 expression makes us feel very interesting, on VT of 12 mL/kg for 8 hours in mice, IL-33 expression of lung tissue homogenates was increase in ventilated mice when compared with control animals. We then comprehensively examined the IL-33 profile, and found a significant increase in IL-33 expression in lung tissue homogenization after VILI. Past studies and the present evidence suggest that IL-33/ST2 activation induces lung injury.

When the IL-33/ST2 pathway is blocked, that may provide an anti-inflammatory protective effect. Recent studies have demonstrated that the IL-33/ST2 pathway contributes to bleomycin-induced fibrotic lung injury [39,40]. Dong et al. indicated that IL-33 is a novel profibrogenic cytokine that signals through ST2 to promote the initiation and progression of pulmonary fibrosis. But, ST2^-/-^ mice could significantly attenuate pulmonary inflammation and fibrosis in a bleomycin-induced lung model. The IL-33/ST2 pathway potentiating could synergistic regulation of the cytokines TGF-β, IL-6, MCP-1, MIP-1α, and TNF-α. However, this study interestingly found that ST2L accumulation in membranes was induced via translocation during VILI in rats. That may contribute to the binding of IL-33 to enhance the consequences of NF-κB activity and lung injury. That also suggests a probable reason that IL-33 secretion into BALF due to VILI was not remarkably increased; i.e., maybe lots of the IL-33 had been bound to the increased levels of ST2L, and thus less IL-33 was present in the serum. Therefore, similar to the above studies, our study indicates that VILI consequences occur via IL-33/ST2 activation. When ST2 receptor is further blocked in VILI may have lung protective effect.

## Conclusions

High-pressure ventilation caused the inflammatory response of IL-33 to be increased in animals, and also caused the IL-33 receptor ST2 to be excessively accumulated in the lungs. Moreover, ST2L-induced membrane translocation resulted in VILI. Therefore, the IL-33/ST2 signaling activated by mechanically responsive lung injury may potentially serve as a new therapy target in efforts to protect the lungs.
